# Prediction Model for Successful Induction of Labor by Fetal Middle Cerebral Artery Pulsatility Index and Obstetric Factors in Term Pregnancy: A Prospective Cohort Study

**DOI:** 10.1155/ogi/7881711

**Published:** 2025-09-04

**Authors:** Vaishali Gautam, Harsha S. Gaikwad, Banashree Nath, Mukesh Shukla, Priti Kumari

**Affiliations:** ^1^Department of Obstetrics and Gynaecology, VMMC and Safdarjung Hospital, Safdarjung Campus, Safdarjung Enclave, New Delhi 110029, Delhi, India; ^2^Department of Obstetrics and Gynaecology, All India Institute of Medical Sciences, Raebareli, Uttar Pradesh, India; ^3^Department of Community and Family Medicine, All India Institute of Medical Sciences, Raebareli, India

**Keywords:** Doppler, induction of labor, prediction model, term pregnancy, ultrasonography

## Abstract

**Objective:** Our study aimed to examine ultrasound and obstetric parameters, explore their interrelationships, and assess their predictive ability in determining the success of labor induction.

**Methodology:** Women with uncomplicated singleton pregnancy at a gestational age of 40 weeks and 3 days with fetal cephalic presentation, having intact fetal membranes and unfavorable Bishop score (BS < 6) were recruited for the study. Ultrasound examination was performed to measure cervical length (CL), estimated fetal weight (EFW), and Doppler velocimetry of fetal cerebral vessels in each patient before induction. We proposed to combine the variables of CL, EFW, BS, and middle cerebral artery pulsatility index (MCA PI) to devise a model for the prediction of successful induction of labor (IOL). IOL was performed with intracervical prostaglandin E2 gel (3 g gel/0.5 mg dinoprostone) applied 6 h apart if needed, not more than 2 doses, followed by oxytocin infusion for up to 6 h. Successful induction was defined as the initiation of active labor at any stage of the induction process.

**Results:** Among the 70 enrolled women, only 29 (41.4%) women responded to induction. CL, BS, and mean value of fetal MCA PI had significant differences in women who responded from those who did not respond to the IOL. The prediction model for the success of induction with the four variables of MCA PI, BS, and CL has a sensitivity of 100% and specificity of 90.2% (AUC 0.982, 95% CI: 0.96–1.00, *p* < 0.001) with the upper cutoff of 0.47. EFW showed to have no effect on the outcome parameter.

**Conclusion:** A model comprising MCA PI, CL, and BS has an excellent prediction value to assess the response to IOL in women at term pregnancy. When a single parameter has to be evaluated, CL is the best maternal factor to predict the success of induction.

## 1. Introduction

Properly timed initiation of labor is a complex process requiring appropriate interactions of the fetal hypothalamo–hypophyseal–adrenal axis, the placenta, fetal membranes, decidua, uterine myometrium, and cervix. Failure to coordinate these interactions impedes labor; several different pathogenic mechanisms may thus result in a continuation of pregnancy beyond term [1]. Induction of labor (IOL) is the procedure of stimulating the uterus to start contractions artificially with the prospect of delivering vaginally [2]. IOL is indicated when the outcomes for the fetus, the mother, or both overweigh the risk of expectant management.

Given the increased incidence of maternal and fetal mortality and morbidity as pregnancy advances beyond term, IOL represents a common intervention in clinical obstetrics accounting for more than 22% of all deliveries [3]. In a retrospective analysis of 171,527 notified births from community child health records in London, the rate of perinatal and infant morbidity increased from 0.35 to 2.12 per 1000 ongoing pregnancies from 37 weeks to 43 weeks of gestation [4].

However, the success of IOL culminating in the delivery of a healthy baby with no complications to the mother is a difficult parameter to predict. Several studies have evaluated the role of demographic, anthropometric, obstetric, and fetal factors such as maternal age, body mass index, gestational age, Bishop score (BS), cervical length (CL), oestriol/estradiol (E3/E2) ratio, fetal weight, and fetal gender as a predictor for response to drugs stimulating labor [5–8]. Marconi in a recent review recommended that the factors influencing the success of induction by different methods should be assessed on an individual basis and any attempt to predict such is far from being certain [9].

Recently, middle cerebral artery (MCA) Doppler changes have been used to predict the onset of labor apart from its use in the evaluation of fetal compromise in fetal growth restriction. Filiberto M. Severi, in their prospective cohort, demonstrated that the MCA pulsatility index (MCA PI) has a nonlinear correlation with gestational age. After an initial increase that extends up to about 30 weeks, the PI shows a decline toward the end of pregnancy. PI reference curves are, in fact, characterized by a parabolic pattern. There is a decline in the resistance of fetal cerebral vessels near term gestation independent of gestational age preceding the onset of labor [10]. Morales-Roselló et al. also demonstrated that there are intense Doppler changes in the cerebral arteries of fetuses near to the onset of labor with a reduction in the resistance in MCA which is not related to gestational age [11]. With the possibility that MCA PI changes can reflect the initiation of labor, it can judiciously be applied to evaluate a potential response to drugs administered to induce labor. Maternal and fetal factors such as CL and fetal weight, respectively, had been used frequently as parameters of a prediction model for successful labor induction [12, 13].

Hence, we propose to combine the variables of CL, estimated fetal weight (EFW), BS, and MCA PI in term pregnancy to evaluate their response to labor-inducing drugs. We aim to combine these parameters to propose a model for the prediction of successful IOL.

## 2. Materials and Methods

### 2.1. Study Setting

The present prospective study was conducted in the Department of Obstetrics and Gynecology at a tertiary hospital in New Delhi, in collaboration with the Department of Radiology for a period of 18 months from October 2019 to April 2020 after a written, informed consent was obtained from all the enrolled women. The study was initiated after approval from the Institute Ethical Committee (IEC).

The optimal time to intervene in an uncomplicated pregnancy after the expected date of delivery is weighed against the risks and benefits of continuation. NICE recommends offering IOL between 41 and 42 completed weeks to avert the risks of prolonged pregnancy [3]. WHO also proposes IOL for women who have reached 41 weeks [2]. The Federation of Obstetric and Gynecological Society of India (FOGSI), however, does not define the upper limit of gestation for IOL but specifies that labor should be induced for a low-risk pregnancy only after 39 weeks [14]. Our institution where the study was conducted adopts its protocol to induce labor in low-risk pregnancies at 40 weeks and 3 days of gestation.

### 2.2. Study Population

Women with uncomplicated singleton pregnancies with a gestational age of 40 weeks and 3 days who have failed to enter active labor spontaneously requiring IOL with fetal cephalic presentation having intact fetal membranes and unfavorable BS (BS < 6) were recruited for the study. Women with pregnancy contraindicated for vaginal delivery, complicated with fetal growth retardation; those requiring caesarean delivery for any maternal and fetal indication; pregnancy complicated with any medical disorders such as gestational HTN, eclampsia, pre-eclampsia, heart disease, renal disease, intrahepatic cholestasis of pregnancy, severe anemia, and gestational diabetes mellitus; and those women with twin pregnancy and congenital anomalies and chromosomal anomalies in fetus were excluded from the study. Women with fetal weight > 4 kgs, low-lying placenta, CL less than 2.5 cm, and decreased liquor (AFI < 5 cm) as well as any woman showing abnormal cardiotocography tracing or willing to opt out of the study before the complete cycle of induction were excluded from the study. Those pregnant females whose fetuses were having abnormal Doppler velocimetry study values were also excluded from the study to avoid bias, as these cases would require intervention that could influence the study outcomes.

### 2.3. Sample Size

In the study conducted by Vannuccini et al. [15] on MCA Doppler to predict the failure of induction, the observed sensitivity and specificity of MCA PI were 71.7% and 90.9% respectively. Taking these values as a reference, the sample size required with a desired precision of 15%, 80% power of study, and 5% level of significance was estimated to be approximately 70 patients.

### 2.4. Study Procedure

Eligible pregnant females were recruited according to the inclusion and exclusion criteria described above. Demographic and clinical characteristics for each patient were collected. Gestational age was determined from the last menstrual period and the same was verified from an early ultrasound scan from the first trimester. The ultrasound scan was performed for all patients just before the IOL. The interval time was not recorded and it varied from 5–30 min depending on logistic and technical considerations. Modified BS was also calculated in each patient before induction.

#### 2.4.1. Ultrasound Examination

Doppler color flow imaging was performed to localize the fetal MCA with the 3.5-MHz curvilinear transducer of the Phillips HD11XE ultrasound device. For performing the procedure, the fetus should be at rest with minimal movements. An axial view of the fetal head was obtained to image the circle of Willis. The MCA vessels were found with color Doppler lying over the anterior wing of the sphenoid bone near the base of the skull and as a major lateral branch of the circle of Willis. The vessel was enlarged to occupy 50% or more of the screen to visualize the artery in its entire length. Spectral trace was obtained from MCA near its origin from the internal carotid artery with a sample volume of 4 mm and keeping the angle of insonation between 0° and 30°. PI was measured both manually as well as in auto mode for three consecutive cardiac cycles and the average value was obtained [16].

Biparietal diameter (BPD), head circumference (HC), abdominal circumference (AC), and femoral length (FL) were measured and fetal weight was determined from an ultrasound machine formula devised by Hadlock et al. [17]. CL was measured by a transabdominal probe with a partially filled maternal bladder. Internal os is located at a point where the fornices of the cervix merge to show a flattened T‐shape or a V‐shaped notch. External os is located near the cervical canal where it meets the vagina [18]. CL was measured by tracing the curved canal from internal to external os [19]. Supplementary ultrasound measurements of Doppler flow parameters such as MCA resistive index and S/D ratio as well as umbilical artery pulsatility, resistive index, and cerebroplacental ratio as well as amniotic fluid volume were also determined as part of the normal obstetric management protocol. The amniotic fluid assessment was performed in all women by adding the anterior–posterior diameters of the deepest fluid pocket, which is free of the umbilical cord or any fetal parts.

#### 2.4.2. IOL

Women included were induced by intracervical prostaglandin E2 gel (Primigyn 3 g/0.5 mg gel) according to the hospital protocol. The dinoprostone gel manufactured by Bharat Serums and Vaccines Limited headquartered in Mumbai, India, was provided free of cost by the Ministry of Health in the hospital. The gel was applied 6 h apart if needed, not to exceed 2 doses. Patients were allowed for ambulation only 30 min after insertion. Patients were observed for temperature, vitals, respiratory rate, uterine activity, and bleeding per vaginum hourly for 4–6 h after insertion of the gel [14]. The second dose was omitted in case of the onset of active labor or abnormal cardiotocography. Patients with favorable BS were augmented with oxytocin. However, any such augmentation was performed only after 6 h of the last dose of dinoprostone. Hence, the entire induction protocol included 2 doses of intracervical dinoprostone gel followed by oxytocin infusion up to 6 h. Successful IOL was defined as the initiation of active labor at any stage of the induction process.

#### 2.4.3. Outcome Measure

The outcome measure will be the onset of labor, which is defined as the establishment of three or more regular uterine contractions each persisting for ≥ 40 s during a period of 10 min with dilatation of cervix ≥ 4 cm. Accordingly, the response to induction will be graded as successful or failed induction.

### 2.5. Statistical Analysis

Analysis of data was performed using the statistical software package SPSS Version 25. The normality of data was tested by the Kolmogorov–Smirnov test. Quantitative variables were correlated using the independent *t-*test/Mann–Whitney test and qualitative variables by the chi-square test/Fisher's exact test. Using the best cutoff values indicated by the ROC analysis, the specificity and sensitivity with their respective 95% confidence bounds and area under the curve were calculated. The probability equation was derived by the multivariable linear regression model where independent variables considered were fetal MCA PI, CL, EFW, and BS, whereas the dependent variable was the success of IOL. The probability of successful induction was calculated from the logistic regression equation for each patient. The statistical significance was achieved when *p* < 0.05.

## 3. Results and Observations

**Figure 1:**
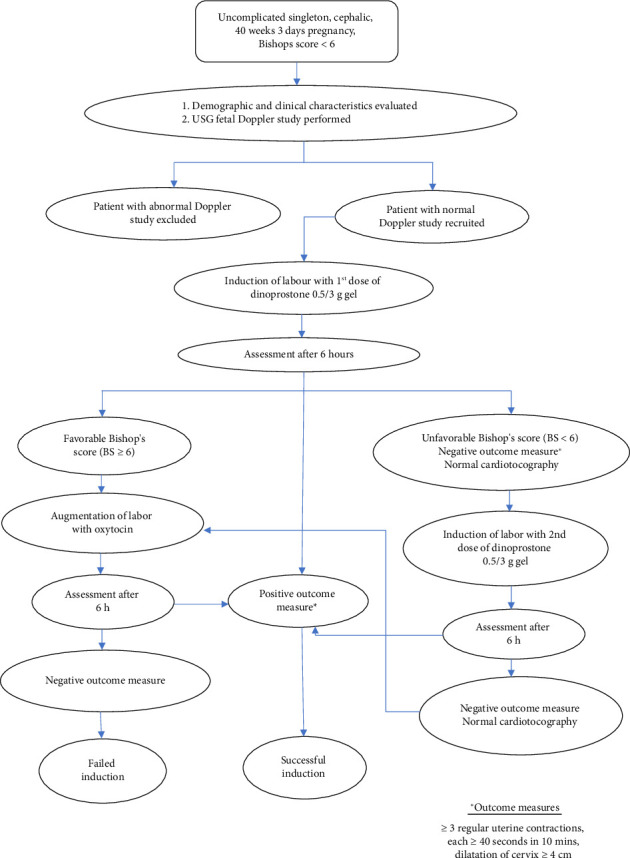
A study methodology flowchart representing the sequence of steps involved in conducting the study.

Among the 70 enrolled women, 29 (41.4%) women responded, while 41 (58.5%) women did not respond to induction. The flowchart for the recruitment of patients and study procedure is presented in Figure 1. The demographic, obstetric, ultrasound, and fetal characteristics in responders and nonresponders are presented in Table 1. Women with young age (*p* = 0.008) and hailing from rural populations (*p* ≤ 0.05) responded favorably to IOL. There was a significant difference in response to induction with relation to different ultrasound parameters such as CL (*p* ≤ 0.05) and amniotic fluid index (*p* ≤ 0.05).

The Doppler flow values of mean MCA PI (*p* ≤ 0.05) and CPR (*p* = 0.017) had significant differences in women who responded from those who did not respond to IOL. The umbilical artery PI, however, was comparable in both the groups.

**Figure 2:**
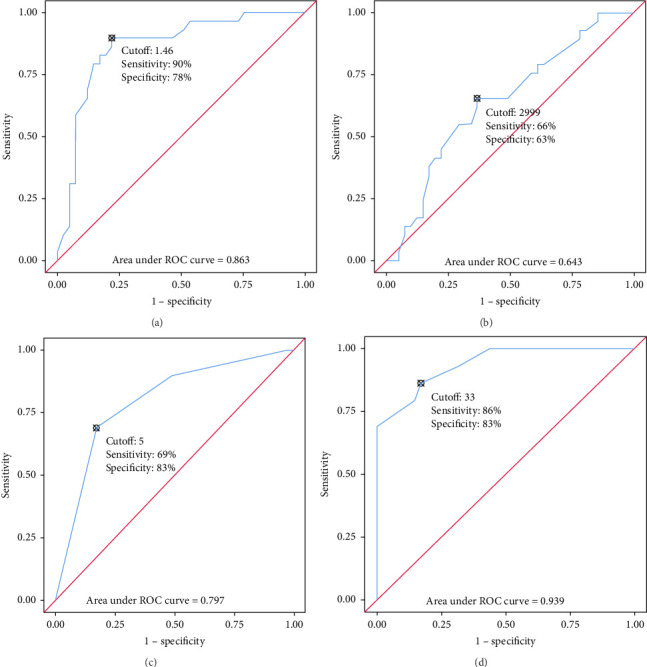
ROC curve analysis showing diagnostic performance of (a) MCA PI, (b) EFW (gm), (c) Bishop score, and (d) cervical length (mm) in predicting the outcome of induction.

Male babies (*p* < 0.05) had a high chance of failure of induction. Receiver operating curve analysis depicting the diagnostic efficacy of (A) MCA PI, (B) EFW (gm), (C) BS, and (D) CL (mm) in predicting the outcome of induction is presented in Figure 2. The cutoff points for different parameters along with their diagnostic efficacy are presented in Table 2. The predictive model for the probability of the successful induction was estimated using a regression model with the four variables of MCA PI, EFW (in gm), modified BS, and CL.

(1) $$\begin{array}{c} Y_{s}\;\left(\text{successful induction}\right)=2.59-0.57\times \text{MCA PI}-0.73\times \text{CL}+0.11\times \text{BS}. \end{array}$$

**Figure 3:**
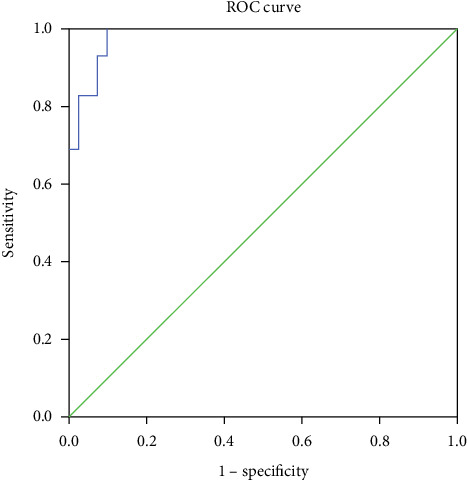
ROC curve analysis of the estimated model probability combining cervical length, Bishop's score, EFW, and MCA PI for the prediction of success of induction.

The Arabic numerals are mathematical constants evaluated using a regression model. EFW is expressed in kilograms and CL is expressed in centimeters. The mathematical constant for the variable of EFW was found to have a nonsignificant association (*p* > 0.05) for the outcome parameter, i.e., the probability for successful IOL. This predictive model for successful IOL was found to have a sensitivity of 100% and a specificity of 90.2% (AUC: 0.982, 95% CI: 0.96–1.00, *p* < 0.001) with an upper cutoff of 0.47 (*Y*_*s*_) (Figure 3). Multinomial logistic regression was performed to adjust for potential covariates, viz., age, BMI, parity, and occupation. However, except for age (AOR = 1.18, 95% CI: 1.02–1.38, *p* = 0.025), no other factor was found to be significantly associated with the success of induction.

## 4. Discussion

A lot of biochemical and structural changes are evident in the uterine body and cervix ahead of the onset of labor. There is an increase in the collagenolytic activity, and alteration in the content of water and proteoglycan in the cervix as pregnancy approaches the term [20]. There is also a decline in cerebral vascular impedance with the onset of labor near-term gestation [10]. These changes can be identified to devise predictive parameters to evaluate the outcome of IOL.

Our study revealed that young mothers and those living in rural areas have a higher chance of delivering after induction. We also found fetal MCA PI was significantly low in women who responded favorably to IOL. We devised a model encompassing the three parameters, viz., MCA PI, CL, and BS to envision the possibilities of success of IOL in women at term pregnancy which revealed excellent prediction value.

The possibility of an older mother responding less favorably to IOL [21] and developing labor dystocia [22] has been proven by different studies. The impairment of the myometrial function with aging due to increased rigidity of the cervix and reduced elasticity of the birth canal has been speculated for such outcomes [23]. When considered discretely, CL was the best parameter to predict the chances of successful induction (AUC: 0.939, 95% CI: 0.888–0.989, *p* < 0.001) among all the four parameters. At a cutoff value of ≤ 33 mm, it predicted success of IOL with a sensitivity of 86% and a specificity of 83%. There is variation in cutoff values of CL in different studies [12, 24]. This is presumably due to differential assumptions of the endpoint of the success of induction. Most of the studies defined successful induction in patients as parturition by vaginal route within a period of 24 h. Since ripening of the cervix can be performed by different methods some exceeding 24 h, setting a 24-h endpoint for delivery may present challenges in ensuring accurate assessment. Although CL alone has shown to be an excellent predictor of labor induction outcomes, the model integrating all three parameters offers significantly higher accuracy and reliability, as evidenced by the ROC curve estimates.

The next best parameter to predict the success of IOL was MCA PI. At a cutoff of MCA PI ≤ 1.46, it predicts a positive response to IOL with a sensitivity of 90% and a specificity of 78%. Our finding of cutoff value is very near to Vannuccini et al. [15], who included only nulliparous women with singleton pregnancies. We included primigravidas and multigravidas almost in equal proportions and hence depicting a more practical representation of the general population. Vannuccini et al. [15] demonstrated that women who carry a fetus having MCA PI lower than 0.74 MoM have significantly lower intervals for the onset of spontaneous labor than those women whose values are higher than 0.74 MoM evaluated between 36^+0^ and 39^+6^ weeks of gestation. The response of induction with lower MCA PI in the subjects in our study at 40^+3^ (PPV = 74.3%, NPV = 91.4%, LR+: 4.08, LR−: 0.13) weeks of gestation reinforces the findings of Filiberto M. Severi et al. [10] that vascular impedance decreases and blood flow velocity increases with advancing gestation. When this sensitive parameter is combined with obstetric parameters already proven to have high sensitivity to predict the success of induction [25], the model is expected to perform accurately for the prediction of the same. Hence, we propose a model encompassing all the three parameters, viz., MCA PI, CL, and BS to anticipate the success of the labor onset which is shown to have excellent potential (AUC: 0.982, 95% CI: 0.960–1.000, *p* < 0.001) for the same. It was observed that the fetal weight was a redundant parameter in the prediction model showing nonsignificant association with the outcome variable. The success or failure of IOL in relation to fetal weight has been controversial with research depicting diverse outcomes. Vrouenraets et al. [26] reported that the risk for caesarean delivery is elevated in nulliparous women who had been induced to initiate labor if birth weight was more than 3.5 kgs (aOR = 1.66, 95% CI: 1.12–2.47) or more than 4 kgs (aOR = 2.38, 95% CI: 1.45–3.91). However, Cheng et al. [27] in their cohort study discovered that a fetus anticipated to have a birth weight of 4 kgs had a higher probability of delivering vaginally with IOL rather than expectant management (OR = 1.25, 95% CI: 1.17–1.33 at 39 weeks). A similar favorable outcome was reported by a large randomized controlled trial by Boulvain et al. [28]. All these studies assumed vaginal delivery as the endpoint of the success of IOL. We found fetal weight contributed none to deciding the success of IOL. The reason that our success was defined by the onset of the active phase of labor and not by vaginal delivery could possibly explain the observation.

Unfortunately, there were a total of three neonatal deaths and one stillbirth in our study. The three neonatal deaths in our study were due to suspected inborn errors of metabolism in one, sudden unexplained death in the second on the eighth day of life at home, and sepsis in the third. The stillbirth occurred in a fetus with cord prolapse. The occurrence of three neonatal deaths and one stillbirth is deeply unfortunate and was thoroughly analyzed. However, these adverse outcomes appeared to be coincidental and could not be attributed to any identifiable shortcomings or deficiencies in our study's design, methodology, or execution. These events underscore the complex and unpredictable factors that can influence neonatal outcomes, many of which were beyond the scope of our study.

Cervical elastography is currently being investigated for its potential role in predicting labor induction outcomes. This diagnostic approach can measure the stiffness or elasticity of the cervix with the potential for prediction of the readiness of the cervix for labor and the likelihood of a successful induction. Artificial intelligence, particularly machine learning algorithms, has the potential to improve the prediction of labor induction outcomes by analyzing vast amounts of clinical data to identify patterns and factors that influence the success or failure of induction. These advancements can help healthcare providers in future to make better-informed decisions about the timing and methods of labor induction, ultimately improving maternal and fetal outcomes.

The strength of our study remains in the inclusion of women of all categories irrespective of parity and cervical condition of women at term gestation exactly at 40^+3^ weeks of gestation, thus reflecting the results which can be applied to the general population. We assumed the endpoint of success of induction as the beginning of an active phase of labor accompanied by adequate contractions and not vaginal delivery. Vaginal delivery may not be warranted even after reaching the active phase of labor and the course of labor beyond it may be altered or modified by innumerable factors including obstetric, maternal, and fetal, and hence equating the success of IOL with vaginal delivery may not be an accurate representation of the clinical entity.

There were several limitations of our study. The use of the model is not advised for high-risk pregnancies or cases involving gestations far from the term. High-risk pregnancies and preterm or postterm gestations often involve unique and complex clinical factors that may not be adequately addressed by the model, thereby limiting its applicability and effectiveness in these specific contexts. Second, variations in expertise among individuals performing ultrasound examinations may lead to interobserver differences, which could contribute to discrepancies in results. Third, the study overlooks the impact of fetal head malrotation on its engagement in the pelvis, which may, consequently, affect the outcome of the induction process. Fetal head malrotation may prevent the head from aligning correctly with the birth canal. Fourth, a potential limitation of the study is its small sample size, which may influence the generalizability and robustness of the findings.

To conclude, a model comprising the three parameters, viz., MCA PI, CL, and BS has excellent prediction value to assess the response to IOL in women at term pregnancy. When a single parameter has to be evaluated, CL is the best maternal factor to predict the success of induction. The accuracy and reliability of the model, however, is superior to this parameter assessed in isolation.

## Figures and Tables

**Table 1:** Demographic, obstetric, ultrasound, and fetal characteristics in responders and nonresponders.

Parameters	Outcome of induction		*p* value
	Responder (*n* = 29)	Nonresponder (*n* = 41)	
Demographic factors			
Mean age	24.41 ± 2.65	26.80 ± 3.86	0.008
Parity			
Primigravida	12 (41.4%)	25 (61.0%)	0.106
Multigravida	17 (58.6%)	16 (39.0%)	
Residence			
Urban	8 (27.6%)	35 (85.4%)	< 0.05
Rural	21 (72.4%)	6 (14.6%)	
BMI	26.95 ± 3.55	28.17 ± 2.68	0.106
Obstetric factors			
Cervical length (mm)			
Median (IQR)	30 (29–32)	36 (34–38)	< 0.05
Modified Bishop's score			
Median (IQR)	5 (4–5)	3 (3–4)	< 0.05
Mode of delivery			
Vaginal	22 (75.9%)	0 (0.0%)	< 0.05
LSCS	7 (24.1%)	41 (100.0%)	
Indication of LSCS			
Failed IOL	0 (0.0%)	35 (85.4%)	< 0.05
Other fetal–maternal indication	7 (100.0%)	6 (14.6%)	
Ultrasound parameters			
MCA PI (mean [S/D])	1.26 (0.24)	1.55 (0.18)	< 0.05
MCA S/D (mean [S/D])	2.82 (0.27)	3.21 (0.31)	< 0.05
CPR (mean [S/D])	1.80 (0.41)	2.03 (0.33)	0.017
AFI (mean [S/D])	12.17 (2.25)	8.27 (1.90)	< 0.05
Fetal parameters			
Baby gender			
Male	3 (10.3%)	34 (82.9%)	< 0.05
Female	26 (89.7%)	7 (17.1%)	
EFW (gm)			
Mean (SD)	2962.97 (197.40)	3065.71 (265.40)	0.068
Fetal outcome			
Live birth	26 (89.7%)	40 (97.6%)	0.310
Early neonatal death	2 (6.9%)	1 (2.4%)	
Stillbirth	1 (3.4%)	0 (0.0%)	

Abbreviations: AFI, amniotic fluid index; CPR, cerebroplacental ratio; IQR, interquartile range; MCA PI, middle cerebral artery pulsatility index; MCA S/D, middle cerebral artery systolic/diastolic (S/D) ratio; UA PI, umbilical artery pulsatility index.

**Table 2:** Best ultrasound parameters to predict the outcome of induction.

Best parameter in terms of AUROC	Cervical length (mm)
Best parameter in terms of sensitivity	AFI (cm) and MCA PI
Best parameter in terms of specificity	CPR
Best parameter in terms of positive predictive value	Cervical length (mm) and MCA S/D
Best parameter in terms of negative predictive value	AFI (cm) and MCA PI
Best parameter in terms of diagnostic accuracy	Cervical length (mm)

Abbreviations: AFI, amniotic fluid index; AUROC, area under the receiver operating characteristic curve; CPR, cerebroplacental ratio; MCA PI, middle cerebral artery pulsatility index; MCA S/D, middle cerebral artery systolic/diastolic (S/D) ratio.

## Data Availability

The data that support the findings of this study are openly available in FIGSHARE at https://doi.org/10.6084/m9.figshare.27247956.v2. Citation: Nath, Banashree (2024). Masterchart.xlsx. figshare. Dataset. https://doi.org/10.6084/m9.figshare.27247956.
